# Supplementary material for: A Benchmark of Modern Statistical Phasing Methods
Source: bioRxiv. 2026 Apr 23:2025.06.24.660794. Preprint. [Version 3] doi: 10.1101/2025.06.24.660794 (PMC13131814; doi:10.1101/2025.06.24.660794)
Supplement: 1 [file NIHPP2025.06.24.660794V3-supplement-1.pdf]

bioRxiv preprint doi: https://doi.org/10.1101/2025.06.24.660794; this version posted April 23, 2026. The copyright holder for this preprint (which was not certified by peer review) is the author/funder, who has granted bioRxiv a license to display the preprint in perpetuity. It is made available under aCC-BY 4.0 International license.

Appendix A Supplementary Figures

bioRxiv preprint doi: https://doi.org/10.1101/2025.06.24.660794; this version posted April 23, 2026. The copyright holder for this preprint (which was not certified by peer review) is the author/funder, who has granted bioRxiv a license to display the preprint in perpetuity. It is made available under aCC-BY 4.0 International license.

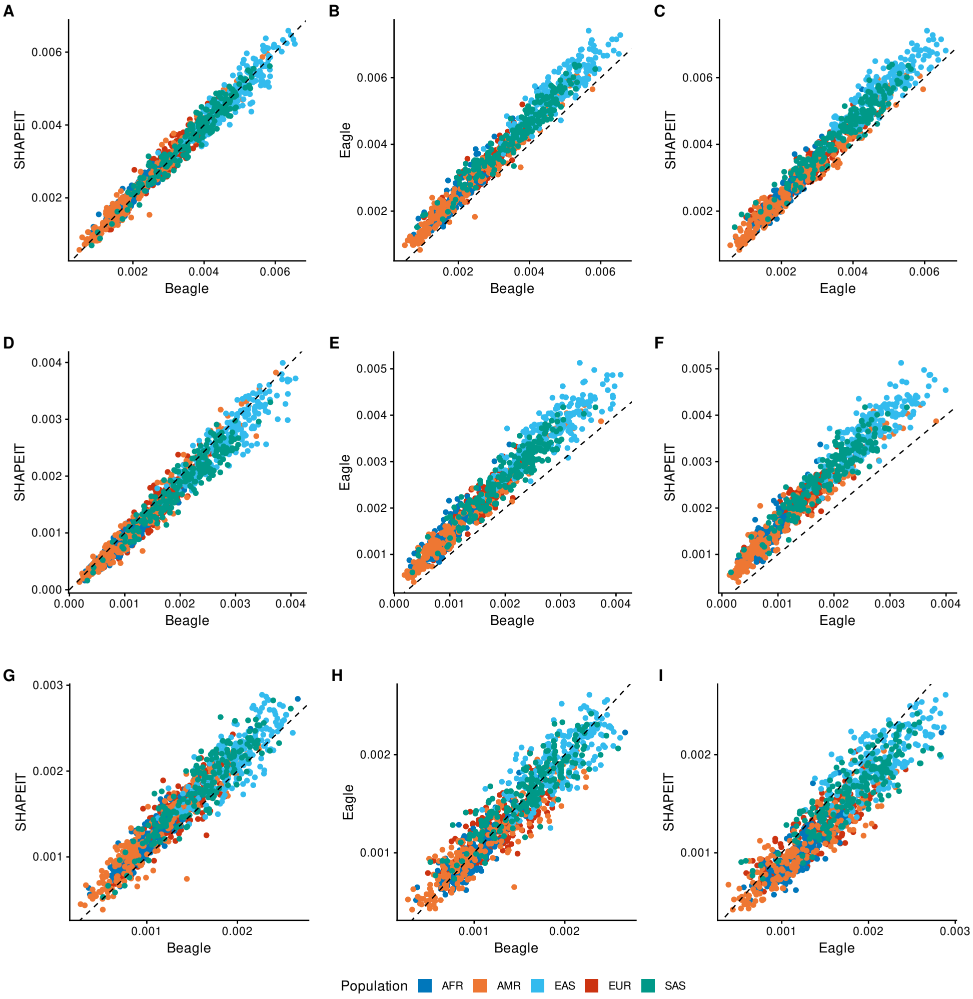

Fig. A1: Error rates observed in each synthetic X chromosome diploid across each pair of methods. Rates are computed as the total number of errors divided by the number of heterozygous positions in each synthetic diploid. Each point represents one synthetic diploid with the x- and y-position determined by the total number of errors observed in each method when comparing the inferred haplotypes to the original male X chromosome haplotypes sampled to generate the synthetic diploid.

bioRxiv preprint doi: https://doi.org/10.1101/2025.06.24.660794; this version posted April 23, 2026. The copyright holder for this preprint (which was not certified by peer review) is the author/funder, who has granted bioRxiv a license to display the preprint in perpetuity. It is made available under aCC-BY 4.0 International license.

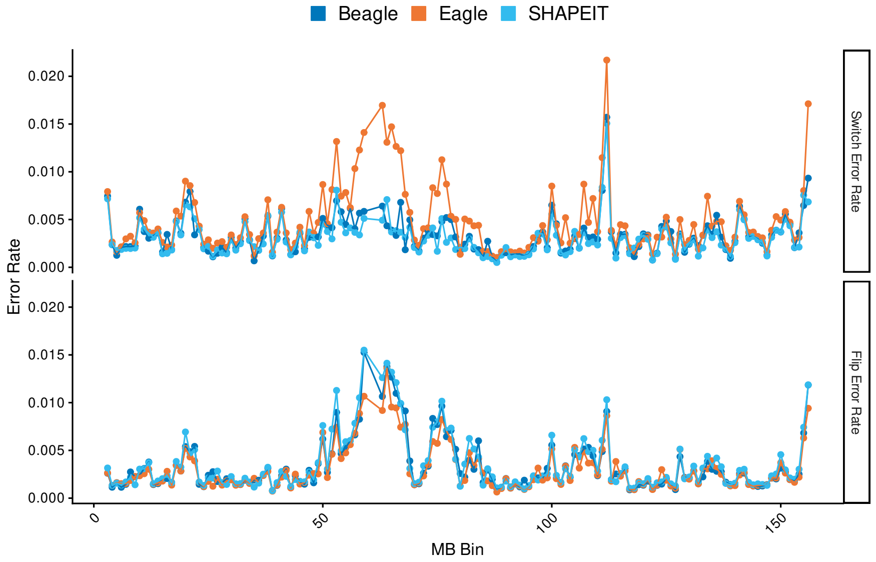

Fig. A2: Average switch and flip error rates are shown in 1-Mb non-overlapping windows across the X chromosome. Each point on the line represents the mean error rate for all 1,000 synthetic diploids within that specific genomic bin. For both switch and flip errors, the rates for each method are highly correlated across the chromosome. However, for switch errors, Eagle (yellow line) exhibits several localized spikes where its error rate is markedly elevated compared to both Beagle and SHAPEIT.

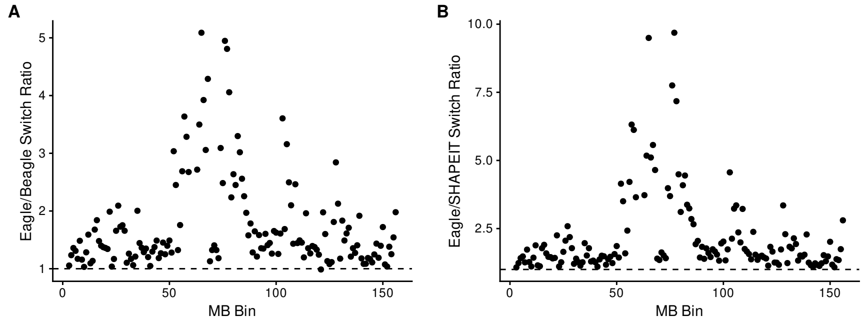

Fig. A3: Enrichment of Eagle switch error rates in MB bins relative to Eagle and SHAPEIT. **(A)** Ratio of Eagle switch error rates to Beagle. On average each bin's average switch rate for Eagle was 1.75 times that of Beagle. **(B)** Ratio of Eagle switch error rates to SHAPEIT. On average each bin's average switch rate for Eagle was 2.18 times that of Beagle.

bioRxiv preprint doi: https://doi.org/10.1101/2025.06.24.660794; this version posted April 23, 2026. The copyright holder for this preprint (which was not certified by peer review) is the author/funder, who has granted bioRxiv a license to display the preprint in perpetuity. It is made available under aCC-BY 4.0 International license.

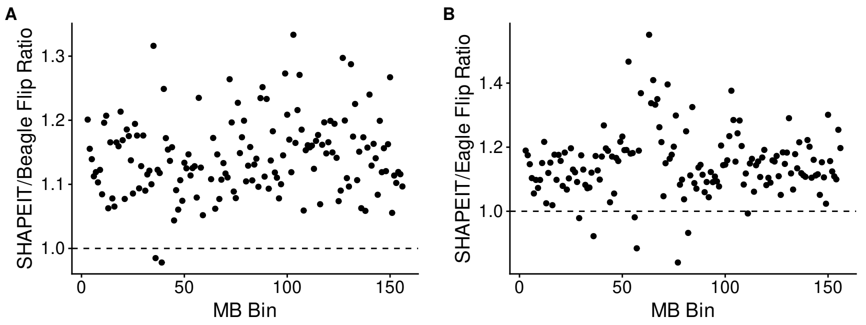

Fig. A4: Enrichment of SHAPEIT flip error rates in MB bins relative to Beagle and Eagle. **(A)** Ratio of SHAPEIT flip error rates to Beagle. On average each bin's average flip rate for SHAPEIT was 1.14 times that of Beagle. **(B)** Ratio of SHAPEIT switch error rates to Eagle. On average each bin's average switch rate for SHAPEIT was 1.15 times that of Eagle.

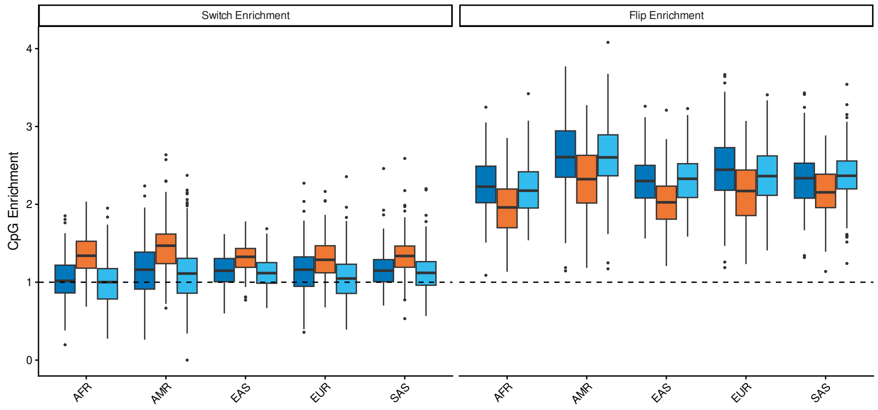

Fig. A5: Distributions of switch error and flip error enrichment observed in chromosome X synthetic diploids for each method by population. For each sample, enrichment is computed by dividing the fraction of errors occurring at CpG sites by the fraction of heterozygous sites at CpG sites. Across methods, both switch ($F(8, 1990) = 7.49, p < 6.88 \times 10^{-10}$) and flip ($F(8, 1990) = 4.23, p < 4.76 \times 10^{-5}$) error rates were found to vary across populations.

bioRxiv preprint doi: https://doi.org/10.1101/2025.06.24.660794; this version posted April 23, 2026. The copyright holder for this preprint (which was not certified by peer review) is the author/funder, who has granted bioRxiv a license to display the preprint in perpetuity. It is made available under aCC-BY 4.0 International license.

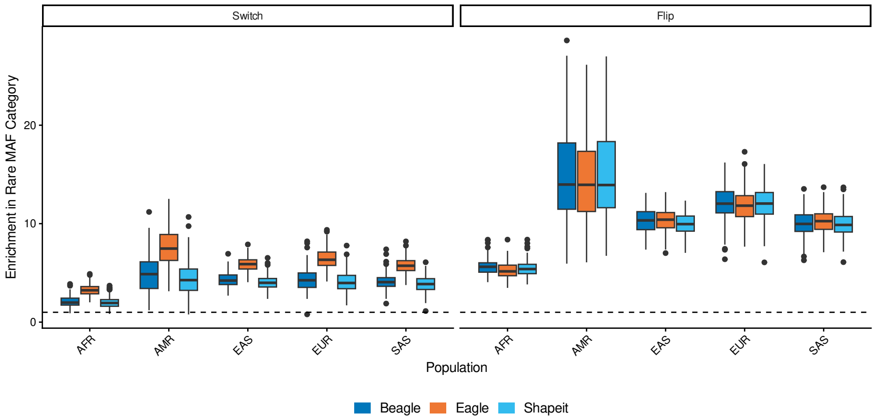

Fig. A6: Enrichment of phasing errors at rare variants by population. For each sample, enrichment is computed by dividing the fraction of errors occurring at rare ($maf <$ 0.05) variants by the fraction of heterozygous sites at rare variants. Across methods, both switch ($F(8, 1990) = 51.42, p < 2.2 \times 10^{-16}$) and flip ($F(8, 1990) = 13.92, p <$ 2.2×10^{-16}) error rates were found to vary across populations.

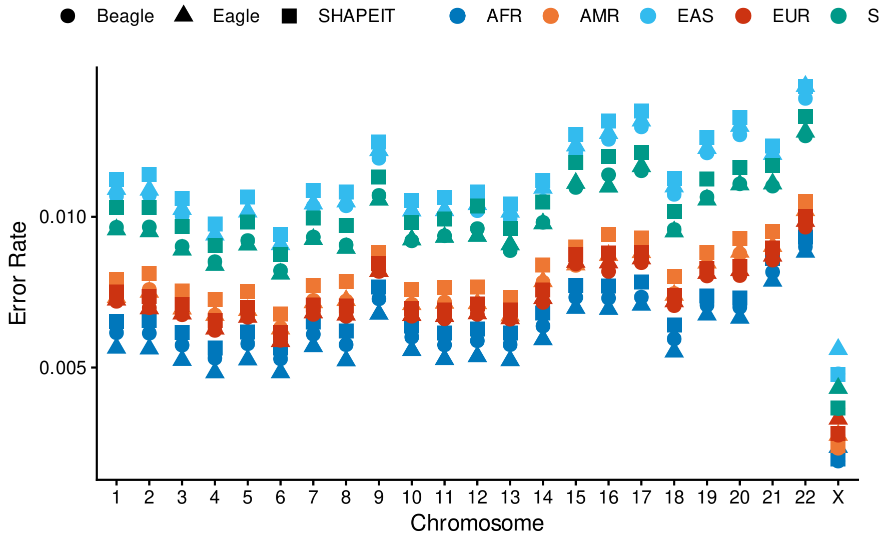

Fig. A7: Phasing error rates observed across the genome, comparing trio-derived autosomal error rates to synthetic X-diploid rates. Phasing method is indicated by point shape, while 1kGP populations are indicated by color.

bioRxiv preprint doi: https://doi.org/10.1101/2025.06.24.660794; this version posted April 23, 2026. The copyright holder for this preprint (which was not certified by peer review) is the author/funder, who has granted bioRxiv a license to display the preprint in perpetuity. It is made available under aCC-BY 4.0 International license.

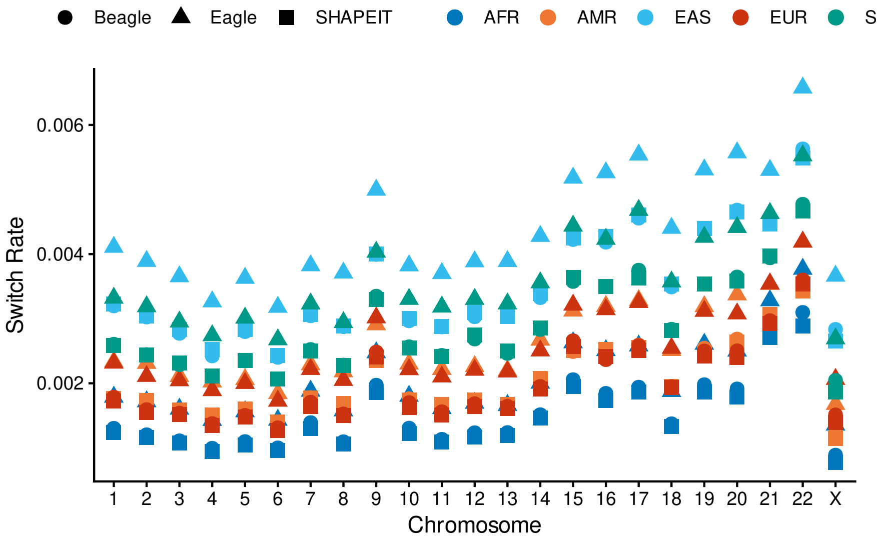

Fig. A8: Mean switch error rates (single switch events) per chromosome. Phasing method is indicated by point shape, while 1kGP populations are indicated by color. Autosomal rates reflect trio proband re-phasing without parental context, provided for comparison against synthetic X-diploid results.

bioRxiv preprint doi: https://doi.org/10.1101/2025.06.24.660794; this version posted April 23, 2026. The copyright holder for this preprint (which was not certified by peer review) is the author/funder, who has granted bioRxiv a license to display the preprint in perpetuity. It is made available under aCC-BY 4.0 International license.

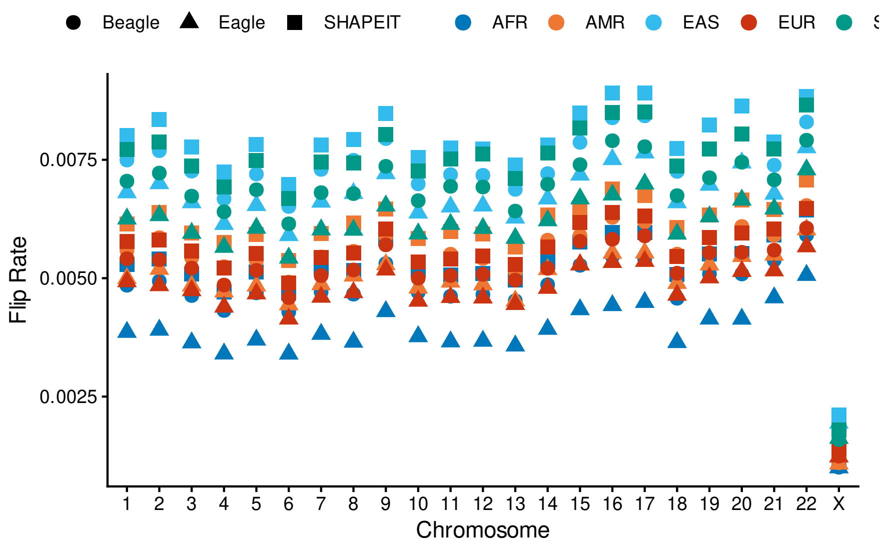

Fig. A9: Mean flip error rates (double switch events) per chromosome. Phasing method is indicated by point shape, while 1kGP populations are indicated by color. Autosomal rates reflect trio proband re-phasing without parental context, provided for comparison against synthetic X-diploid results.

bioRxiv preprint doi: https://doi.org/10.1101/2025.06.24.660794; this version posted April 23, 2026. The copyright holder for this preprint (which was not certified by peer review) is the author/funder, who has granted bioRxiv a license to display the preprint in perpetuity. It is made available under aCC-BY 4.0 International license.

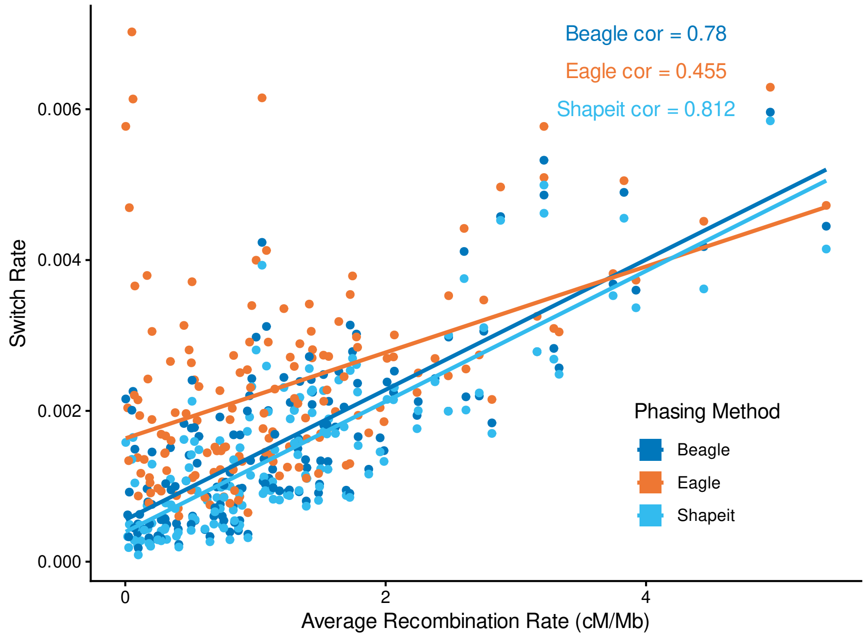

Fig. A10: Average switch error rates and recombination rate values in non-overlapping MB bins in chromosome X. Switch error rates are observed to be correlated with average recombination rates in non-overlapping MB bins in chromosome X (Beagle: $r = 0.78, p < 0.001$; Eagle: $r = 0.45 (p < 0.001)$; SHAPEIT: $r = 0.81 (p < 0.001)$).

bioRxiv preprint doi: https://doi.org/10.1101/2025.06.24.660794; this version posted April 23, 2026. The copyright holder for this preprint (which was not certified by peer review) is the author/funder, who has granted bioRxiv a license to display the preprint in perpetuity. It is made available under aCC-BY 4.0 International license.

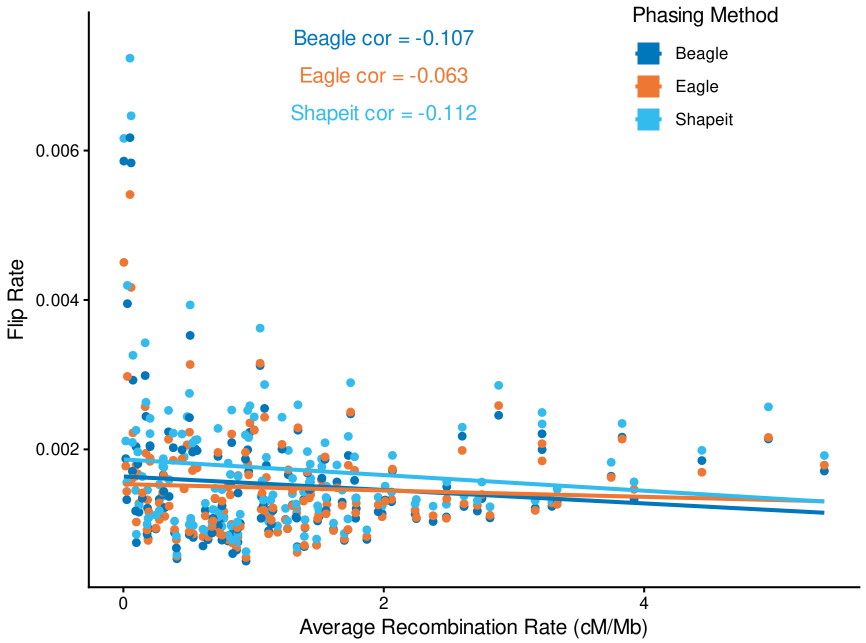

Fig. A11: Average flip error rates and recombination rate values in non-overlapping MB bins in chromosome X. Flip errors are not found to be significantly correlated with recombination rates.

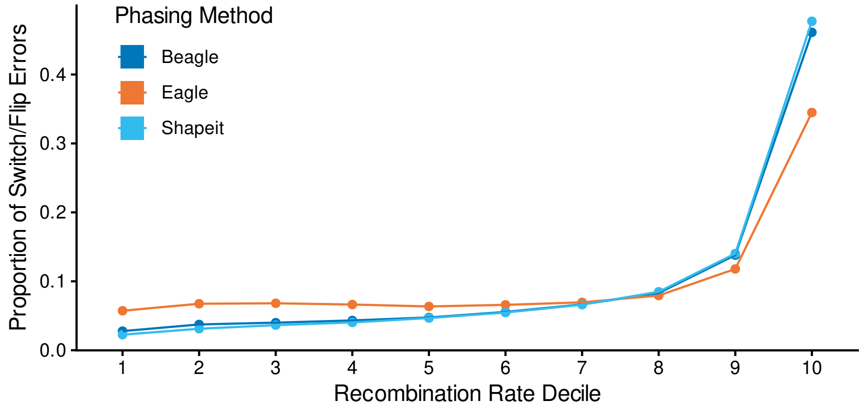

Fig. A12: Proportion of switch errors stratified by recombination rate deciles. Mean proportion of total switch errors occurring at heterozygous sites within each recombination decile. Results are averaged over 1000 synthetic diploids. The top decile (highest recombination rate) contains an enriched proportion of total switch errors (> 10%).

bioRxiv preprint doi: https://doi.org/10.1101/2025.06.24.660794; this version posted April 23, 2026. The copyright holder for this preprint (which was not certified by peer review) is the author/funder, who has granted bioRxiv a license to display the preprint in perpetuity. It is made available under aCC-BY 4.0 International license.

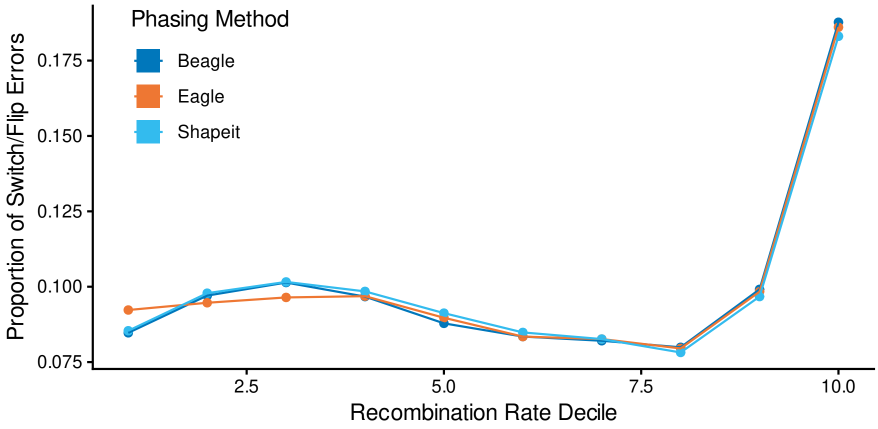

Fig. A13: Proportion of flip errors stratified by recombination rate deciles. Mean proportion of total flip errors occurring at heterozygous sites within each recombination decile. Results are averaged over 1000 synthetic diploids. The top decile (highest recombination rate) contains an enriched proportion of flip errors (> 10%).

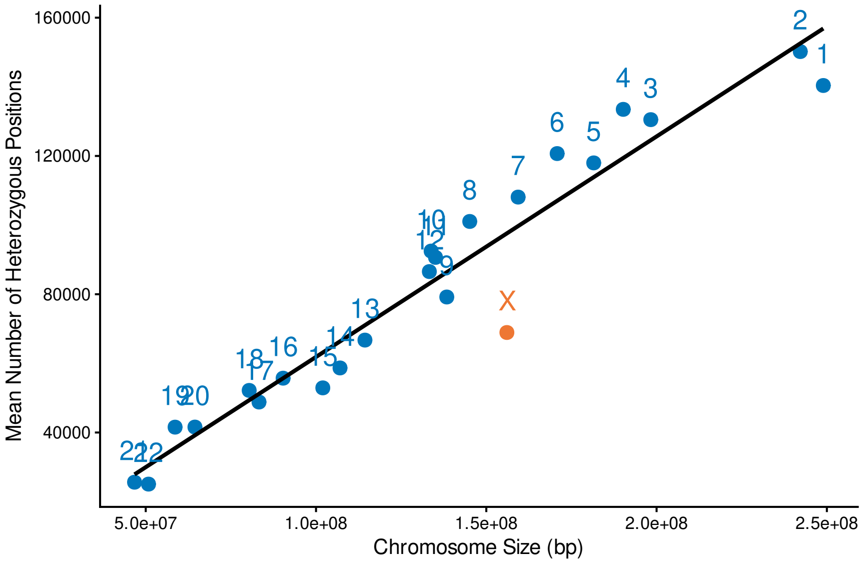

Fig. A14: Relationship between chromosome size and heterozygosity. Mean number of heterozygous sites plotted against physical chromosome size (Mb) for autosomal trio probands and synthetic diploid X-chromosomes. The average number of heterozygous positions in chromosome X synthetic diploids is found to be less than similarly sized autosomes.

bioRxiv preprint doi: https://doi.org/10.1101/2025.06.24.660794; this version posted April 23, 2026. The copyright holder for this preprint (which was not certified by peer review) is the author/funder, who has granted bioRxiv a license to display the preprint in perpetuity. It is made available under aCC-BY 4.0 International license.

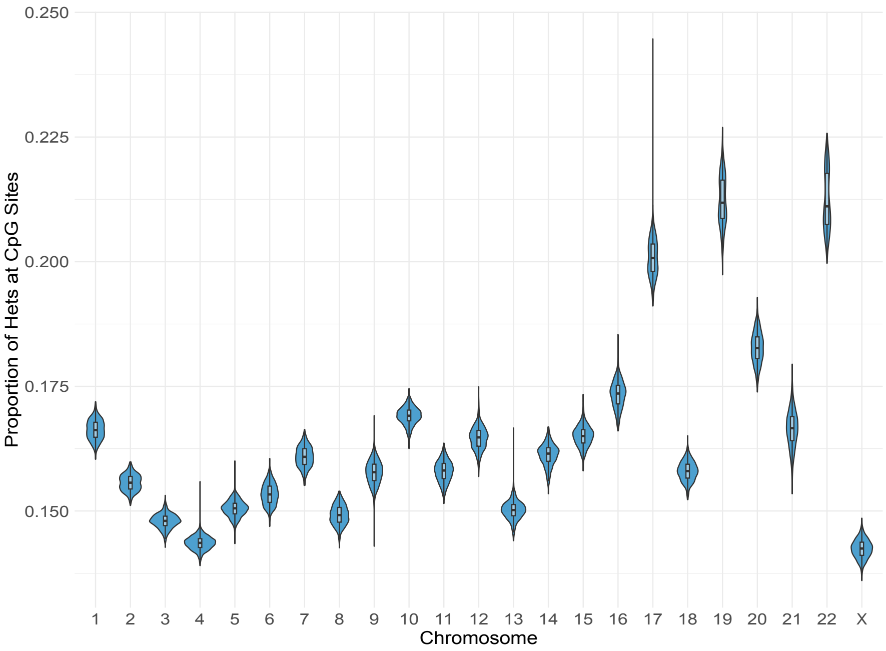

Fig. A15: Distribution of the proportion of heterozygous sites occurring at CpG dinucleotides across all autosomes and chromosome X. Synthetic X-chromosomes show a reduced proportion of CpG-associated heterozygosity relative to the autosomes.

bioRxiv preprint doi: https://doi.org/10.1101/2025.06.24.660794; this version posted April 23, 2026. The copyright holder for this preprint (which was not certified by peer review) is the author/funder, who has granted bioRxiv a license to display the preprint in perpetuity. It is made available under aCC-BY 4.0 International license.

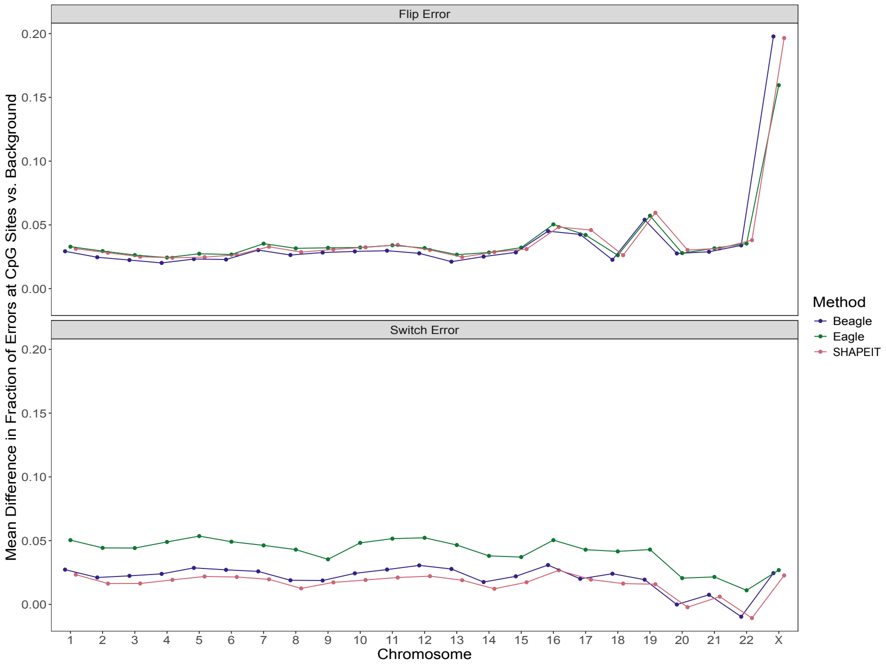

Fig. A16: Enrichment of flips and switch errors at CpG sites across the autosomes (trio re-phase) and X (synthetic diploids). Enrichment is computed as the mean difference between the proportion of errors at CpG sites and the proportion of heterozygous positions at CpG sites.

bioRxiv preprint doi: https://doi.org/10.1101/2025.06.24.660794; this version posted April 23, 2026. The copyright holder for this preprint (which was not certified by peer review) is the author/funder, who has granted bioRxiv a license to display the preprint in perpetuity. It is made available under aCC-BY 4.0 International license.

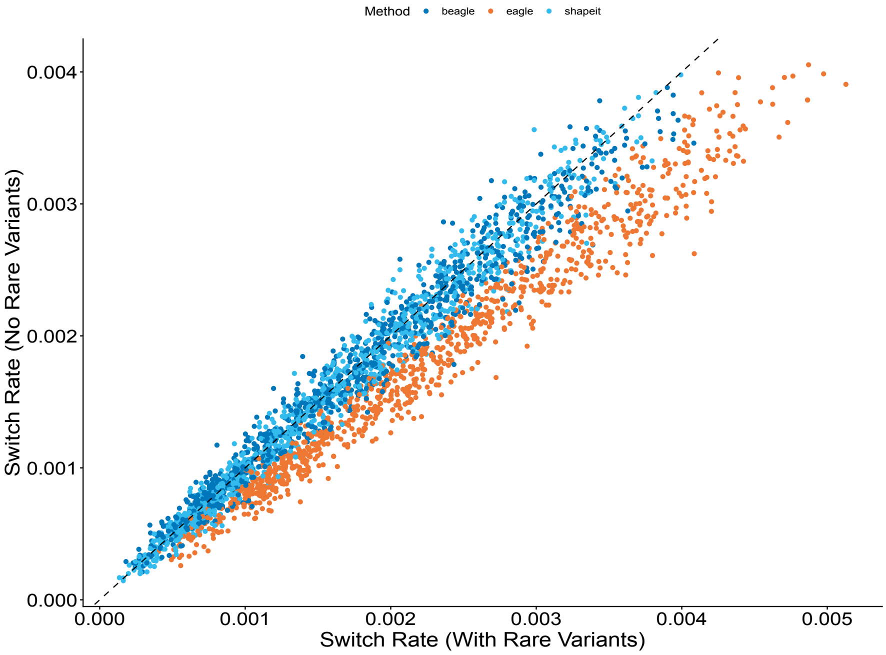

Fig. A17: Switch rates observed in the 1000 synthetic diploids with and without very rare ($maf < 0.001$) variants.

bioRxiv preprint doi: https://doi.org/10.1101/2025.06.24.660794; this version posted April 23, 2026. The copyright holder for this preprint (which was not certified by peer review) is the author/funder, who has granted bioRxiv a license to display the preprint in perpetuity. It is made available under aCC-BY 4.0 International license.

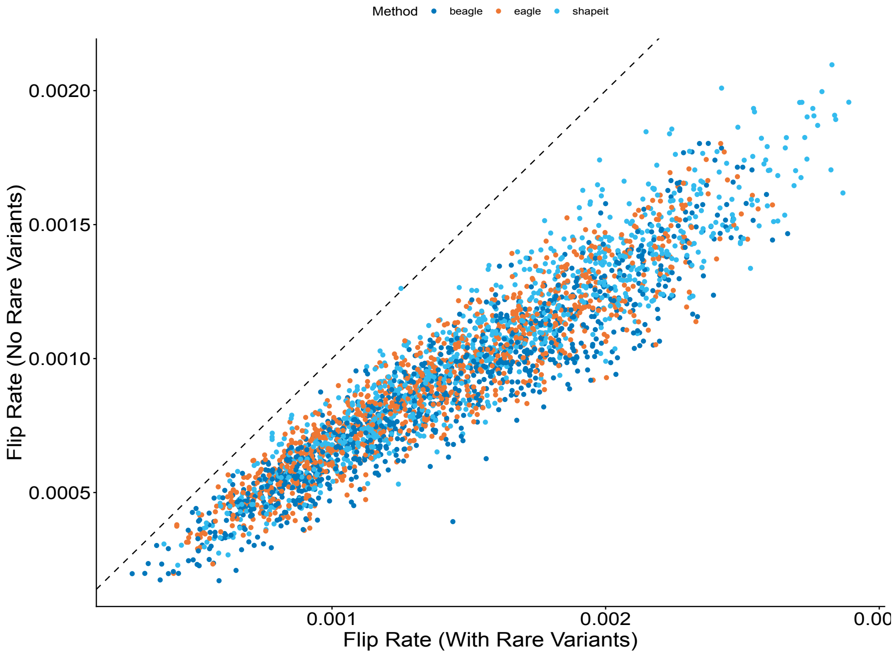

Fig. A18: Switch rates observed in the 1000 synthetic diploids with and without very rare ($maf < 0.001$) variants.

bioRxiv preprint doi: https://doi.org/10.1101/2025.06.24.660794; this version posted April 23, 2026. The copyright holder for this preprint (which was not certified by peer review) is the author/funder, who has granted bioRxiv a license to display the preprint in perpetuity. It is made available under aCC-BY 4.0 International license.

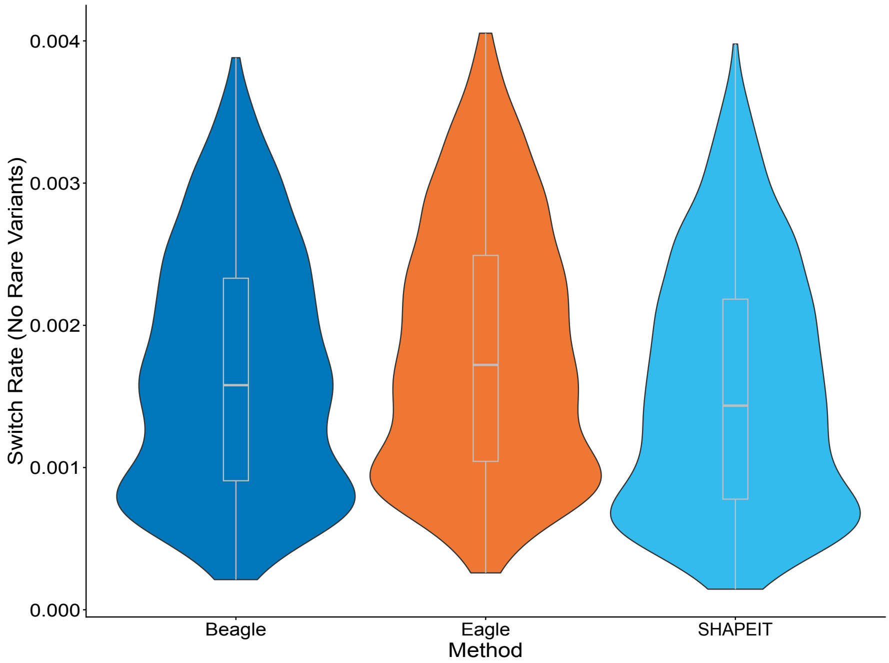

Fig. A19: Distributions of switch error rates by method in the 1000 synthetic X diploids phased with very rare ($maf < 0.001$) variants removed.

bioRxiv preprint doi: https://doi.org/10.1101/2025.06.24.660794; this version posted April 23, 2026. The copyright holder for this preprint (which was not certified by peer review) is the author/funder, who has granted bioRxiv a license to display the preprint in perpetuity. It is made available under aCC-BY 4.0 International license.

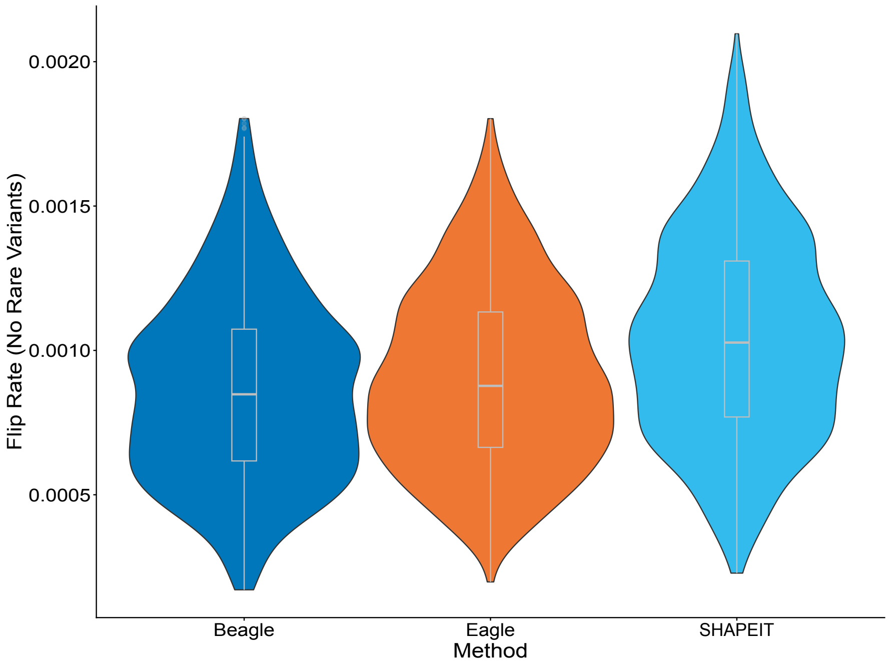

Fig. A20: Distributions of flip error rates by method in the 1000 synthetic X diploids phased with very rare ($maf < 0.001$) variants removed.

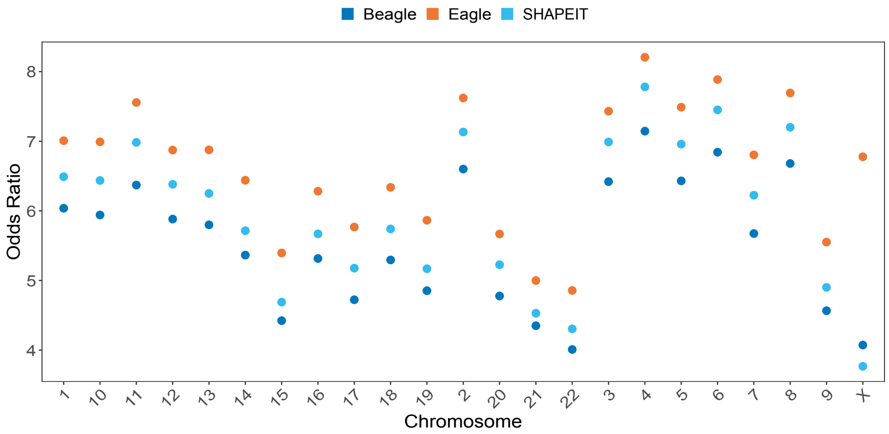

Fig. A21: Odds ratios for switches at rare variants by chromosome and method. Data points represent the ratio calculated from the sum of all observations across all samples ($n = 602$ trio autosomes, $n = 1000$ X synthetic diploids).

bioRxiv preprint doi: https://doi.org/10.1101/2025.06.24.660794; this version posted April 23, 2026. The copyright holder for this preprint (which was not certified by peer review) is the author/funder, who has granted bioRxiv a license to display the preprint in perpetuity. It is made available under aCC-BY 4.0 International license.

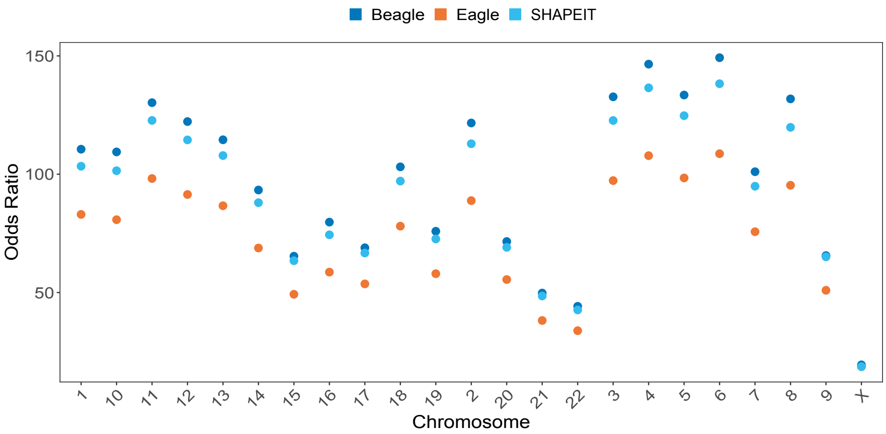

Fig. A22: Odds ratios for flips at rare variants by chromosome and method. Data points represent the ratio calculated from the sum of all observations across all samples ($n = 602$ trio autosomes, $n = 1000$ X synthetic diploids).

bioRxiv preprint doi: https://doi.org/10.1101/2025.06.24.660794; this version posted April 23, 2026. The copyright holder for this preprint (which was not certified by peer review) is the author/funder, who has granted bioRxiv a license to display the preprint in perpetuity. It is made available under aCC-BY 4.0 International license.

Appendix B Supplementary Tables

Error Type	Method	Mean Difference	T	P value	CI Lower	CI Upper
Flip Error	Beagle	0.1977867	110.07309	0	0.1948284	Inf
Flip Error	Eagle	0.1595476	93.97320	0	0.1567524	Inf
Flip Error	SHAPEIT	0.1965362	115.14546	0	0.1937261	Inf
Switch Error	Beagle	0.0244927	32.94071	0	0.0232685	Inf
Switch Error	Eagle	0.0268967	36.95026	0	0.0256983	Inf
Switch Error	SHAPEIT	0.0227155	31.24358	0	0.0215185	Inf

Table B1: T test results comparing the proportion of errors at CpG sites to the background proportion of heterozygous sites at CpG sites.

Category	Rare	Uncommon	Common
Background	0.0602	0.0853	0.855
Beagle	0.216	0.148	0.637
Eagle	0.317	0.142	0.541
SHAPEIT	0.202	0.144	0.654

Table B2: Average proportions of heterozygous positions and switch error locations by minor allele frequency bins in X chromosome synthetic diploids. Each synthetic diploid has a proportion of its heterozygous positions and its errors for each method within each of these bins, and here we present the average of these values over all 1000 synthetic diploids.

bioRxiv preprint doi: https://doi.org/10.1101/2025.06.24.660794; this version posted April 23, 2026. The copyright holder for this preprint (which was not certified by peer review) is the author/funder, who has granted bioRxiv a license to display the preprint in perpetuity. It is made available under aCC-BY 4.0 International license.

Category	Rare	Uncommon	Common
Background	0.0602	0.0853	0.855
Beagle	0.569	0.108	0.323
Eagle	0.56	0.112	0.328
SHAPEIT	0.562	0.112	0.326

Table B3: Average proportions of heterozygous positions and flip error locations by minor allele frequency bins in X chromosome synthetic diploids. Each synthetic diploid has a proportion of its heterozygous positions and its errors for each method within each of these bins, and here we present the average of these values over all 1000 synthetic diploids.

Chromosome	Average Heterozygous	Size	Mean Density
1	140381.27	248,956,422	0.0005638788864
2	150199.66	242,193,529	0.000620163803
3	130503.74	198,295,559	0.0006581273966
4	133478.04	190,214,555	0.0007017235879
5	117995.98	181,538,259	0.0006499785811
6	120689.8	170,805,979	0.0007065900193
7	108102.46	159,345,973	0.0006784135047
8	101055.01	145,138,636	0.0006962653969
9	79201.45	138,394,717	0.0005722866574
10	92489.26	133,797,422	0.0006912633937
11	90662.32	135,086,622	0.0006711421061
12	86556.05	133,275,309	0.0006494530056
13	66732.95	114,364,328	0.0005835119321
14	58679.32	107,043,718	0.0005481808844
15	52914.63	101,991,189	0.0005188156989
16	55717.3	90,338,345	0.0006167624612
17	48818.39	83,257,441	0.0005863546779
18	52180.24	80,373,285	0.0006492236817
19	41554.18	58,617,616	0.0007089025934
20	41571.99	64,444,167	0.0006450853807
21	25626.59	46,709,983	0.0005486319702
22	25074.31	50,818,468	0.0004934094038
X	68930	152,939,905	0.0004506999007

Table B4: The mean number of heterozygous sites per proband (autosomes) and synthetic diploids (X) and chromosome sizes from GRCh38.

bioRxiv preprint doi: https://doi.org/10.1101/2025.06.24.660794; this version posted April 23, 2026. The copyright holder for this preprint (which was not certified by peer review) is the author/funder, who has granted bioRxiv a license to display the preprint in perpetuity. It is made available under aCC-BY 4.0 International license.

	Mean Error Rate		
Chromosome	Beagle	Eagle	SHAPEIT
1	0.0077	0.0075	0.0081
2	0.0077	0.0075	0.0082
3	0.0072	0.0070	0.0077
4	0.0067	0.0066	0.0072
5	0.0072	0.0070	0.0077
6	0.0065	0.0063	0.0069
7	0.0074	0.0073	0.0079
8	0.0073	0.0071	0.0078
9	0.0087	0.0085	0.0092
10	0.0073	0.0072	0.0077
11	0.0073	0.0071	0.0077
12	0.0074	0.0072	0.0079
13	0.0071	0.0070	0.0076
14	0.0078	0.0078	0.0084
15	0.0089	0.0088	0.0094
16	0.0090	0.0089	0.0095
17	0.0091	0.0091	0.0096
18	0.0076	0.0075	0.0081
19	0.0087	0.0086	0.0090
20	0.0089	0.0088	0.0093
21	0.0093	0.0092	0.0098
22	0.0105	0.0105	0.0109
X	0.0031	0.0037	0.0031

Table B5: Mean error rates observed in probands (autosomes) and synthetic diploids (X).

bioRxiv preprint doi: https://doi.org/10.1101/2025.06.24.660794; this version posted April 23, 2026. The copyright holder for this preprint (which was not certified by peer review) is the author/funder, who has granted bioRxiv a license to display the preprint in perpetuity. It is made available under aCC-BY 4.0 International license.

	Mean Switch Rate			Mean Flip Rate		
Chromosome	Beagle	Eagle	SHAPEIT	Beagle	Eagle	SHAPEIT
1	0.0019	0.0025	0.0019	0.0057	0.0050	0.0062
2	0.0018	0.0024	0.0018	0.0059	0.0051	0.0064
3	0.0017	0.0022	0.0017	0.0055	0.0048	0.0060
4	0.0015	0.0021	0.0015	0.0052	0.0045	0.0057
5	0.0017	0.0022	0.0016	0.0055	0.0048	0.0060
6	0.0015	0.0020	0.0014	0.0050	0.0044	0.0054
7	0.0019	0.0025	0.0019	0.0055	0.0048	0.0060
8	0.0017	0.0022	0.0017	0.0056	0.0049	0.0061
9	0.0026	0.0032	0.0025	0.0061	0.0053	0.0066
10	0.0019	0.0024	0.0018	0.0054	0.0048	0.0059
11	0.0017	0.0023	0.0017	0.0055	0.0048	0.0060
12	0.0018	0.0024	0.0018	0.0055	0.0048	0.0060
13	0.0018	0.0024	0.0018	0.0053	0.0046	0.0058
14	0.0021	0.0027	0.0021	0.0057	0.0050	0.0062
15	0.0027	0.0034	0.0027	0.0061	0.0054	0.0067
16	0.0026	0.0034	0.0026	0.0064	0.0055	0.0070
17	0.0028	0.0035	0.0027	0.0064	0.0056	0.0069
18	0.0021	0.0027	0.0021	0.0055	0.0048	0.0060
19	0.0027	0.0034	0.0026	0.0059	0.0052	0.0064
20	0.0028	0.0034	0.0027	0.0061	0.0054	0.0066
21	0.0032	0.0038	0.0032	0.0060	0.0054	0.0065
22	0.0038	0.0045	0.0037	0.0066	0.0060	0.0072
X	0.0017	0.0023	0.0016	0.0014	0.0014	0.0016

Table B6: Mean switch and flip error rates observed in probands (autosomes) and synthetic diploids (X).
